# Influence of a Wearable Fitness Tracker on Time to Return to Baseline Activity Following Abdominoplasty: A Randomized Control Trial

**DOI:** 10.1093/asjof/ojaf135

**Published:** 2025-10-21

**Authors:** Erin N Abbott, Nomongo Dorjsuren, Barite Gutama, Anthony L Hoang, Mariam Saad, Galen Perdikis, Kent Higdon

## Abstract

**Background:**

Postoperative management following abdominoplasty varies widely, particularly regarding improving the time to return to baseline physical activity.

**Objectives:**

The goal of the authors of this randomized controlled trial was to compare the rate of return to baseline activity after abdominoplasty between patients receiving activity reminders from a wearable fitness tracker (actigraphy) and those following standard postoperative activity recommendations.

**Methods:**

Patients undergoing cosmetic abdominoplasties were enrolled between December 2020 and December 2022. Those using actigraphy devices with activity reminders before enrollment were excluded. Patients were randomized to receive a wearable actigraphy monitor either with or without movement reminders. Only those with at least 5 days of preoperative and 7 weeks of postoperative step data were included in the final analysis.

**Results:**

A total of 51 patients were enrolled in the study with a mean age of 44 ± 10.2 years. The attrition rate was 30/51 (59%), with 67% and 52% of the intervention group not completing the study (*P* = .16). Among the 21 patients with complete data, the mean time to return to baseline steps was 5.4 weeks in the control group and 5.6 weeks in the intervention group (*P* = .81). By the end of postoperative Week 8, 87.5% of the control group and 84.6% of the intervention group had reached their baseline (*P* = 1).

**Conclusions:**

Structured reminders through an actigraphy device did not significantly change the time to return to baseline after abdominoplasty. Most patients returned to their baseline steps at the end of the study period regardless of intervention, providing valuable insight into the typical recovery time following abdominoplasty.

**Level of Evidence: 2 (Therapeutic):**

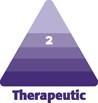

Abdominoplasty is one of the most commonly performed cosmetic procedures in the United States, with an estimated 195,000 abdominoplasties in 2023.^1^ Despite its prevalence, the procedure is associated with a prolonged recovery period. Patients are typically advised to begin ambulation immediately, maintain a flexed position for up to 2 weeks to minimize complications such as seroma or wound dehiscence, and avoid vigorous activity for up to 6 weeks.^2-4^ However, the timeline for resuming physical activity remains largely driven by expert consensus rather than supported by robust clinical evidence, leading to variability in recommendations across different practices. Furthermore, although early mobilization is encouraged, defining an optimal rehabilitation protocol remains challenging because recovery patterns may differ based on surgical technique, patient characteristics, and adherence to postoperative instructions.

Early mobilization, physiotherapy, and compression therapy is recommended in patients undergoing abdominoplasty to optimize recovery and minimize complications.^5,6^ However, most existing research focuses on postoperative complications rather than structured rehabilitation strategies. Additionally, although some studies suggest that gradual return to physical activity may improve postoperative outcomes, there is a lack of standardized guidelines for monitoring or encouraging patient mobility. This variability in postsurgical recommendations contributes to differences in patient adherence and recovery outcomes.^7^

Given these gaps in standardized postoperative activity guidance, further research is needed to determine effective strategies for enhancing recovery following abdominoplasty. In this randomized controlled trial, the authors aim to assess whether structured physical activity reminders can accelerate return to baseline activity levels compared with standard postoperative rehabilitation recommendations. Reminders were delivered through a wrist-worn actigraphy device that objectively monitors physical activity by tracking step count.

## METHODS

This study was approved by the IRB (#190154). Patients scheduled for abdominoplasty between December 2020 and December 2022 were identified through electronic medical records and contacted for enrollment based on predefined inclusion and exclusion criteria. Patients were included if they were undergoing an abdominoplasty for cosmetic indications and excluded if they were using any wearable fitness tracker (actigraphy device) with exercise reminders before the study. After obtaining informed consent, patients were randomized into 2 groups in a 1:1 ratio using a random number generator, with no stratification. All participants were mailed a commercially available actigraphy device, the Fitbit Inspire HR (Fitbit Inc., San Francisco, CA). The devices for the intervention group were programmed to deliver 10 hourly movement reminders starting on postoperative Day 1. These consisted of short haptic vibrations and visual prompts such as “Time to move!” to encourage light activity and did not include specific step targets or directives regarding intensity or pacing. Participants kept the devices after the study concluded.

Procedures were performed by 8 different board-certified plastic surgeons at a single institution. Although technique and postoperative opioid prescribing varied modestly by surgeon based on preference, all patients underwent standard cosmetic abdominoplasty and were discharged on the same day as surgery. All patients received uniform postoperative care instructions to ambulate early as tolerated, maintain a flexed posture at the hips for 2 weeks, sleep with the head of the bed elevated, and refrain from strenuous activity during the initial postoperative period. No participants in either group were specifically instructed to return to their preoperative activity level by any particular timeline. Operative reports were reviewed to extract surgical variables, including concurrent surgeries, plication of the rectus abdominis, transversus abdominis plane (TAP) block use, volume of lipoaspirate removed from the trunk, and number of drains placed. Postoperative prescriptions were also reviewed for duration of opioid prescription and need for refills.

The primary outcome was the time taken to return to baseline step count after surgery. Reaching the baseline step count at any point during follow-up, even if subsequent values fell below it, was considered as having returned to baseline activity. Baseline step count was defined as the mean daily step count recorded over at least 5 preoperative days. This threshold was selected in accordance with previous literature, suggesting that 3 to 5 days of monitoring is sufficient to estimate habitual physical activity using wrist-worn accelerometers, particularly for light or moderate activity levels.^8,9^ For data to be considered meaningfully complete, participants had to establish a baseline step count, miss no more than 4 consecutive days of step data collection, and have step data available through at least 7 weeks postoperatively.

Descriptive statistics were used to summarize patient characteristics, attrition, and step count trends. Kaplan–Meier survival analysis was conducted to estimate time to return to baseline step count, with group comparisons assessed using the log-rank test. Additional comparisons were made using *t*-tests and Fisher's exact tests, with statistical significance set at *P* < .05.

## RESULTS

### Study Enrollment and Attrition

**Figure 1.**
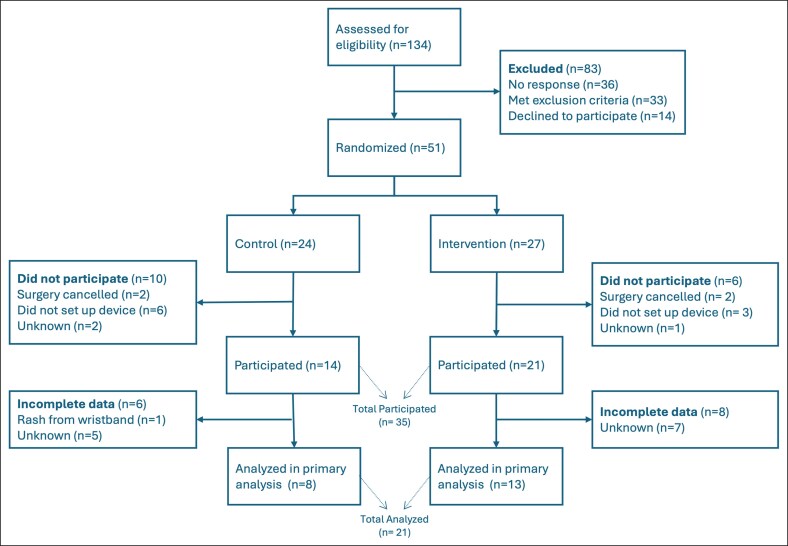
CONSORT diagram showing patient flow through enrollment to analysis. A total of 134 patients were assessed for eligibility. Fifty-one patients were randomized to either the control group (*n* = 24) or intervention group (*n* = 27). Among those who participated, incomplete data led to exclusion from the final analysis in 6 control and 8 intervention group patients. The final analytic sample included 8 control and 13 intervention patients.

A total of 134 patients were assessed for eligibility, with 83 excluded because of no response (*n* = 36, 43.3%), meeting exclusion criteria (*n* = 33, 39.8%), or declining participation (*n* = 14, 16.9%). Ultimately, 51 patients were consented and randomized into either the intervention (*n* = 27) or control (*n* = 24 groups). The overall attrition rate was 16/51 (31%) with dropouts occurring because of surgery cancellation (*n* = 4), failure to set up device (*n* = 9), or unknown reasons (*n* = 3). Among the 35 patients who actively engaged in the study, 21 (60%) provided complete step data and were included in final analysis (Figure 1).

### Baseline Characteristics

All patients were female. The mean age of the enrolled population was 44.2 ± 10.2 years old. Baseline demographics of the randomized intervention and control groups were compared, with no significant differences in age, BMI, comorbidities, or smoking status (Table 1). Similarly, among those who completed the study and were included in further analysis, baseline characteristics remained similar between intervention and control groups. No statistically significant difference was found in the distribution of surgeons between the intervention and control groups (*P* = .52).

**Table 1.** Baseline Characteristics of Patients Randomized to the Intervention vs Control Group

	Control (*n* = 24)	Intervention (*n* = 27)	*P*-value
Age (years)			
Mean ± SD	45.5 ± 10.2	43.0 ± 10.2	.38
BMI (kg/m^2^)			
Mean ± SD	30.9 ± 7.26	30.1 ± 5.60	.66
Diabetes			
No	22 (91.7%)	22 (81.5%)	.42
Yes	2 (8.3%)	5 (18.5%)	
Hypertension			
No	19 (79.2%)	19 (70.4%)	.69
Yes	5 (20.8%)	8 (29.6%)	
Cardiovascular disease			
No	24 (100%)	24 (88.9%)	.24
Yes	0 (0%)	3 (11.1%)	
Smoking status			
Never	18 (75.0%)	18 (66.7%)	.55
Former	6 (25%)	9 (33.3%)	
Current	0 (0%)	0 (0%)	

SD, standard deviation.

### Study Completion

A total of 13/27 (48.1%) of the intervention group and 8/24 (33%) of control group completed the study (*P* = .18). Among those who completed the study, baseline characteristics remained similar between the intervention and control groups (Table 2). To assess potential selection bias, baseline characteristics and surgical factors of patients with complete vs incomplete data were compared. No significant difference in baseline demographics and preoperative characteristics was observed (Table 3). Similarly, surgical complications and procedural factors did not significantly differ between those with complete vs incomplete data. Specifically, there were no significant differences in rectus abdominis plication, TAP block use, and opioid prescribing patterns between groups (Table 4). Although patients underwent a range of concurrent cosmetic procedures (Supplemental Table 1), there were no statistically significant differences between the intervention and control groups or between completers and noncompleters (Supplemental Tables 2  and 3).

**Table 2.** Baseline Characteristics of Intervention and Control Groups Among Study Completers

	Control (*n* = 8)	Intervention (*n* = 13)	*P*-value
Age (years)			
Mean ± SD	51.4 ± 9.44	42.4 ± 10.6	.06
BMI (kg/m^2^)			
Mean ± SD	27.5 ± 7.04	29.7 ± 6.78	.48
Diabetes			
No	7 (87.5%)	12 (92.3%)	1
Yes	1 (12.5%)	1 (7.7%)	
Hypertension			
No	5 (62.5%)	9 (69.2%)	1
Yes	3 (37.5%)	4 (30.8%)	
Cardiovascular disease			
No	8 (100%)	11 (84.6%)	.5
Yes	0 (0%)	2 (15.4%)	
Smoking status			
Never	7 (87.5%)	9 (69.2%)	
Former	1 (12.5%)	4 (30.8%)	
Current	0 (0%)	0 (0%)	.61

SD, standard deviation.

**Table 3.** Baseline Characteristics of Patients With Complete vs Incomplete Data

	Complete (*n* = 21)	Incomplete (*n* = 14)	*P*-value
Age (years)			
Mean ± SD	45.8 ± 10.9	42.7 ± 8.98	.37
BMI (kg/m^2^)			
Mean ± SD	28.9 ± 6.79	30.8 ± 5.19	.36
Diabetes			
No	19 (90.5%)	11 (78.6%)	.37
Yes	2 (9.5%)	3 (21.4%)	
Hypertension			
No	14 (66.7%)	10 (71.4%)	1
Yes	7 (33.3%)	4 (28.6%)	
Cardiovascular disease			
No	19 (90.5%)	13 (92.9%)	1
Yes	2 (9.5%)	1 (7.1%)	
Smoking status			
Never	16 (76.2%)	8 (57.1%)	.28
Former	5 (23.8%)	6 (42.9%)	
Current	0 (0%)	0 (0%)	
Group			
Control	8 (38.1%)	7 (50.0%)	.73
Intervention	13 (61.9%)	7 (50.0%)	

SD, standard deviation.

**Table 4.** Surgical Factors and Complications of Patients With Complete vs Incomplete Data

	Complete (*n* = 21)	Incomplete (*n* = 14)	*P*-value
Concurrent procedure			
No	5 (23.8%)	4 (28.6%)	1
Yes	16 (76.2%)	10 (71.4%)	
Trunk liposuction			
No	7 (33.3%)	1 (7.1%)	
Yes	14 (66.7%)	13 (92.9%)	.11
Rectus diastasis repair			
No	1 (4.8%)	2 (14.3%)	.55
Yes	20 (95.2%)	12 (85.7%)	
TAP block used			
No	15 (71.4%)	12 (85.7%)	.43
Yes	6 (28.6%)	2 (14.3%)	
Amount of lipoaspirate (mL)			
Mean ± SD	436 ± 263	452 ± 475	.92
Missing	7 (33.3%)	2 (14.3%)	
Number of drains placed			
Mean ± SD	1.48 ± .512	1.64 ± 0.497	.35
Days of opioids prescribed			
Mean ± SD	3.76 ± 2.43	2.93 ± 1.38	.21
Required additional opioids			
No	18 (85.7%)	13 (92.9%)	.64
Yes	3 (14.3%)	1 (7.1%)	
Any postoperative complication			
No	18 (85.7%)	9 (64.3%)	.22
Yes	3 (14.3%)	5 (35.7%)	
Surgical-site infection			
No	21 (100%)	12 (85.7%)	.15
Yes	0 (0%)	2 (14.3%)	
Seroma			
No	19 (90.5%)	14 (100%)	.51
Yes	2 (9.5%)	0 (0%)	
Hematoma			
No	20 (95.2%)	12 (85.7%)	.55
Yes	1 (4.8%)	2 (14.3%)	
Wound dehiscence			
No	20 (95.2%)	14 (100%)	1
Yes	1 (4.8%)	0 (0%)	
Reoperation			
No	20 (95.2%)	12 (85.7%)	.55
Yes	1 (4.8%)	2 (14.3%)	

SD, standard deviation; TAP, transversus abdominis plane.

### Time to Return to Baseline Activity

**Figure 2.**
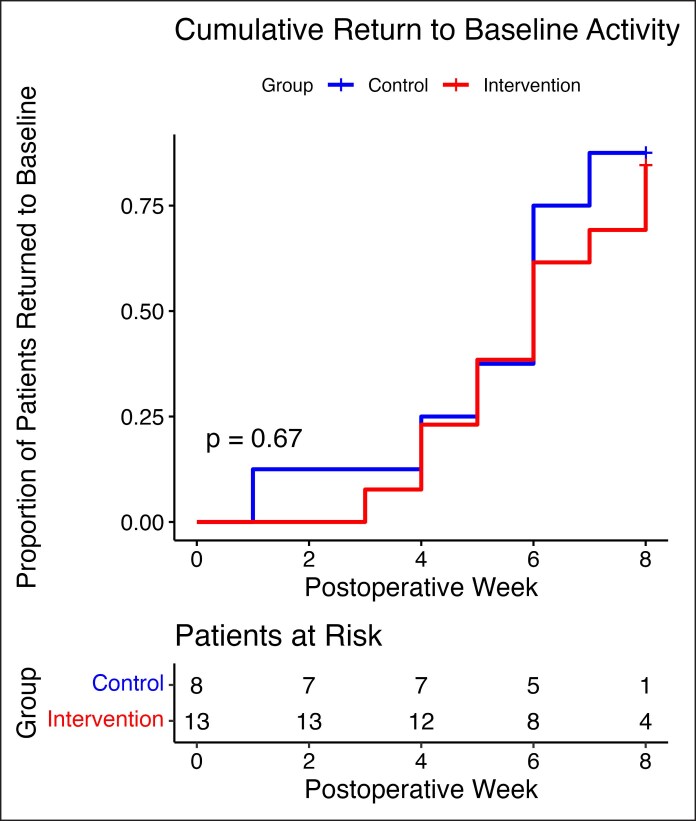
Kaplan–Meier survival curve for cumulative proportion of patients who returned to baseline steps, defined as the 7-day average step count and stratified by study group. The control group is shown in blue and the intervention group in red. “Patients at Risk” reflects the number of participants who had not yet returned to baseline at the start of each postoperative week.

The mean time to return to baseline step count was 5.38 ± 2.13 weeks in the control group and 5.60 ± 2.06 weeks in the intervention group (*P* = .81). The median time to baseline steps was 6 weeks in both groups. Of those who completed the study, nearly all patients had returned to baseline by the end of postoperative Week 8 with 7/8 (87.5%) of the control group and 11/13 (84.6%) of the intervention group patients meeting their preoperative average step count at the time of study completion. This difference was not statistically significant (*P* = 1). A Kaplan–Meier survival curve illustrates the proportion of participants returning to baseline over time, showing no significant difference between groups (*P* = .67; Figure 2).

### Weekly Step Trends and Variability

**Figure 3.**
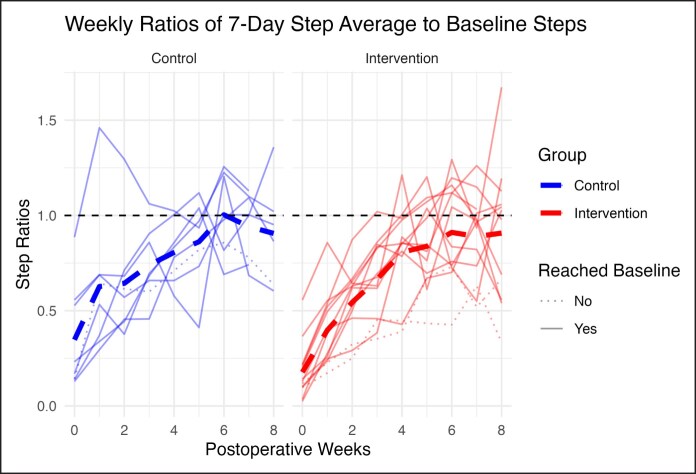
Individual patient trajectories of weekly 7-day step averages normalized to preoperative baseline activity. The control group is shown in blue (left) and the intervention group in red (right). Solid lines indicate patients who returned to baseline activity during follow-up, whereas dotted lines indicate patients who did not. Thick dashed lines represent group means. The horizontal dashed line at 1.0 marks preoperative baseline activity.

The mean preoperative step baseline was 8351 ± 2871 steps (*n* = 21). By Week 4, the average 7-day daily step count was 6788 ± 3110 steps (ratio of 0.82). By Week 8, 86.4% (18/21) of participants had reached to their baseline, preoperative step count, with an average 7-day daily step count of 7703 ± 2954 steps (ratio of 0.92; Figure 3). Although most participants achieved baseline activity during the 8-week follow-up period, there was variability in whether they maintained it, with some subsequently experiencing a decrease in step counts. The normalized maximum postoperative 7-day average step count was 1.16 ± 0.19 for the control group and 1.14 ± 0.25 for the intervention group (*P* = .58). Although the means were similar, the intervention group showed greater interpatient variability, as reflected by a higher coefficient of variation (22.3% vs 16.1%; Table 5). There was no significant difference between groups in normalized maximum postoperative activity (Welch's *t*-test *P* = .125; Wilcoxon *P* = .143).

**Table 5.** Normalized Ratio of Maximum Postoperative 7-Day Average Step Count Relative to Preoperative Baseline

	Mean^a^	Standard deviation (±SD)	Coefficient of variance (CV)	Range
Control	1.16	0.18	16.1	0.9-1.5
Intervention	1.10	0.24	22.0	0.6-1.7

SD, standard deviation.

^a^Values represent unitless ratios calculated by dividing each patient's maximum postoperative 7-day average daily step count by their preoperative average daily step count. A value of 1.0 indicates a return to baseline activity; values >1.0 reflect step counts above baseline.

## DISCUSSION

The authors of this study investigate the use of a structured physical reminder for patients, delivered through a wearable actigraphy monitor, to enhance postabdominoplasty recovery. There was no statistically significant difference in the average postoperative maximum steps taken between the intervention and control groups. However, the intervention group exhibited the greater variability and outliers, with some patients demonstrating strong improvements up to 1.6 times their mean maximum step baseline whereas others only achieved up to 0.6 times their baseline. In contrast, the control group displayed less variability with most patients reaching a mean maximum step value close to their baseline. A greater proportion of patients in the intervention group completed the study compared with the control group. This may suggest that the reminder feature modestly improved adherence, even if it did not impact overall activity levels.

The absence of statistically significant differences in postoperative activity between the control and intervention group may be attributed to behavioral and psychosocial changes following abdominoplasty. Abdominoplasty has been shown to improve quality of life, reduce psychosocial distress, and promote activity.^10-12^ In our study, 86% of patients who completed the study had returned to their baseline step count by 8 weeks postoperatively, regardless of group assignment. Although most patients achieved their preoperative step count by Week 8, our analysis demonstrated that some subsequently fell below baseline activity levels. This pattern suggests that meeting baseline activity may be transient for certain individuals, highlighting the importance of ongoing monitoring beyond initial recovery milestones. Given that abdominoplasty patients are generally highly motivated to stay active postoperatively, it is possible that both groups have remained similarly engaged in physical activity. Thus, additional wearable actigraphy monitor reminders seem redundant. More significant effects of wearable actigraphy monitor reminders may be observed in certain surgical patients who have low preoperative and postoperative activity, such as cancer surgery patients and elderly patients. Notably, patients with complete and incomplete data did not differ significantly in baseline demographics, operative factors, postoperative opioid prescribing, or complication rates, suggesting minimal evidence of selection bias among the analyzed patients.

Although there is limited the literature directly assessing the time required to return to preoperative activity level after abdominoplasty, most patients are discharged home within 1 to 2 days, with adequate postoperative support such as pain control and activity recommendations.^13,14^ Early discharge to home facilitates a quicker return to baseline activity, as reflected in our study, where full baseline functional recovery was achieved within 5.5 to 5.6 weeks, compared with the 3 months it could take for patients undergoing cancer-related surgery.^15,16^ The findings suggest that abdominoplasty patients are inherently active and recover quickly, diminishing the marginal benefits of wearable actigraphy monitor reminders in this highly motivated population.

The greater variability observed in the intervention group may reflect differences in individual responses to wearable actigraphy monitor reminders. Highly motivated individuals may have responded positively by significantly increasing their activity levels, whereas others may have found the reminders discouraging and responded by reducing their activity level. This subgroup of patients who experienced decreased activity because of wearable actigraphy monitor reminders is particularly noteworthy and warrants further investigation. This aligns with previous findings that passive interventions may not universally promote recovery, and underscores the need for more personalized, adaptive strategies when leveraging wearable technology.^17,18^ It is concerning that reminders may have a negative psychological impact on some patients, potentially leading to distress and reduced activity levels. Another unintended consequence of providing real-time actigraphy feedback may be the development of a false sense of security, leading patients to believe they are recovering adequately without the need for additional effort or adherence to postoperative guidelines. Therefore, wearable actigraphy monitor reminders should be used with caution, and patients should be closely monitored to identify those who may not benefit from, or could even be adversely affected by, such interventions. Although wearable activity trackers were used in this study as part of a research protocol, they are not routinely incorporated into postoperative care for abdominoplasty patients at this institution. Based on our findings, we do not anticipate benefit for most patients; however, targeted use in higher-risk or less active individuals may still hold value.

The use of a wearable actigraphy monitor, specifically a Fitbit, has been validated across various surgical disciplines as a tool for both preoperative and postoperative patient evaluation. It has been shown to enhance postoperative recovery, with studies reporting up to 41% of cancer patients using Fitbit returned to preoperative physical activity levels within 3 months postsurgery.^19^ Moreover, wearable actigraphy monitors have demonstrated potentials as an adjunct for evaluating preoperative functional capacity.^15^ Additionally, the step counts from wearable actigraphy monitors have been explored as predictors of readmission following cancer surgery, benchmarks for recovery after total knee arthroplasty, and tools for monitor recovery after appendectomy in pediatrics and head and neck surgery.^16,20-22^ However, these studies are observational studies and do not assess the effect of an intervention utilizing wearable actigraphy monitors’ built-in features to enhance postoperative recovery. Although similar studies have investigated the impact of Fitbit reminders on other plastic surgery procedures, this study is the first study to evaluate the impact of activity reminders through a wearable actigraphy monitor on recovery after abdominoplasty.^23^

This study has several limitations. Its small sample size weakens the strength of analysis that was performed. Although the study was randomized, participants were not blinded to their intervention status, which may have influenced activity behaviors or engagement. The study also carries a high risk of attrition bias, with 45% in the intervention group and 67% in the control group failing to complete it. This substantially reduced the analyzable population and may have affected the results. However, since there were no significant differences in baseline characteristic, operative factors, or surgical complications between patients with complete and incomplete data, the risk of bias from attrition was somewhat mitigated. Still, self-selection bias is a possible factor because participants knew they were being monitored and may represent a more motivated subset of abdominoplasty patients. The generalizability of this study is likely not representative of the general population. Similarly, another limitation of this study is that patients underwent a variety of concurrent procedures, which could plausibly influence recovery. Although the distribution was similar across groups, the potential impact of additional surgery on activity recovery cannot be fully excluded.

Furthermore, the observed heterogeneity in response to reminders suggests that psychological and behavior factors, such as intrinsic motivation, device fatigue, or performance anxiety, may influence recovery trajectories, but these were not formally assessed. There are also potential unmeasured confounders related to both device and person factors. Although the actigraphy device was validated for step tracking, there may have been variability in device wear time, placement, and syncing behavior that could affect data accuracy and completeness. Likewise, although step count offers an objective measure of mobility, it does not capture more strenuous or functional outcomes that patients often prioritize, such as returning to work, lifting, or driving. We were unable to evaluate the relationship between preoperative exercise habits and study attrition, because this information was not recorded. Additionally, other factors like baseline fitness level and muscle composition, home environment, pain perception, or availability of support systems were not formally assessed but may impact recovery trajectories and adherence to the study protocol. In future studies, these factors should be systematically captured using a broader range of functional metrics or validated recovery questionnaires. Larger sample sizes will be necessary to more reliably evaluate the impact of pre-, intra-, and postoperative factors, including actigraphy with or without reminders, on recovery outcomes. The researchers of future studies could assess tailored interventions such as personalized activity goals and feedback, alongside baseline fitness analysis and long-term recovery tracking, particularly for patients at risk for delayed recovery.

## CONCLUSIONS

This is the first randomized controlled trial to examine the impact of wearable activity reminders in abdominoplasty patients. Most patients returned to baseline activity within 6 to 8 weeks, with no significant difference between intervention and control groups in time to recovery or normalized maximum activity. The intervention group showed greater variability, suggesting individualized responses to reminders. The lack of significantly higher rates of return to baseline activity with actigraphy-based reminders may reflect that abdominoplasty patients tend to recover quickly, regardless of additional prompting. These findings suggest that standard postoperative instructions may be sufficient for this generally motivated and active population.

## Supplemental Material

This article contains supplemental material located online at https://doi.org/10.1093/asjof/ojaf135.

## Supplementary Material

ojaf135_Supplementary_Data
