# Supplementary material for: Influence of a Wearable Fitness Tracker on Time to Return to Baseline Activity Following Abdominoplasty: A Randomized Control Trial
Source: Aesthet Surg J Open Forum. 2025 Oct 21;7:ojaf135. doi: 10.1093/asjof/ojaf135 (PMC12635626; doi:10.1093/asjof/ojaf135)

Supplemental Table 1. Distribution of concurrent procedures performed across the study cohort

| Procedure | Number |
| --- | --- |
| Mastopexy | 18 |
| Fat grafting to breast | 9 |
| Augmentation mastopexy | 5 |
| Secondary breast augmentation | 5 |
| Primary breast augmentation | 3 |
| Mammoplasty | 3 |
| Abdominal hernia repair | 2 |
| Brachioplasty | 1 |
| Blepharoplasty | 1 |

Supplementary Table 2. Concurrent procedures among patients randomized to the intervention and control groups


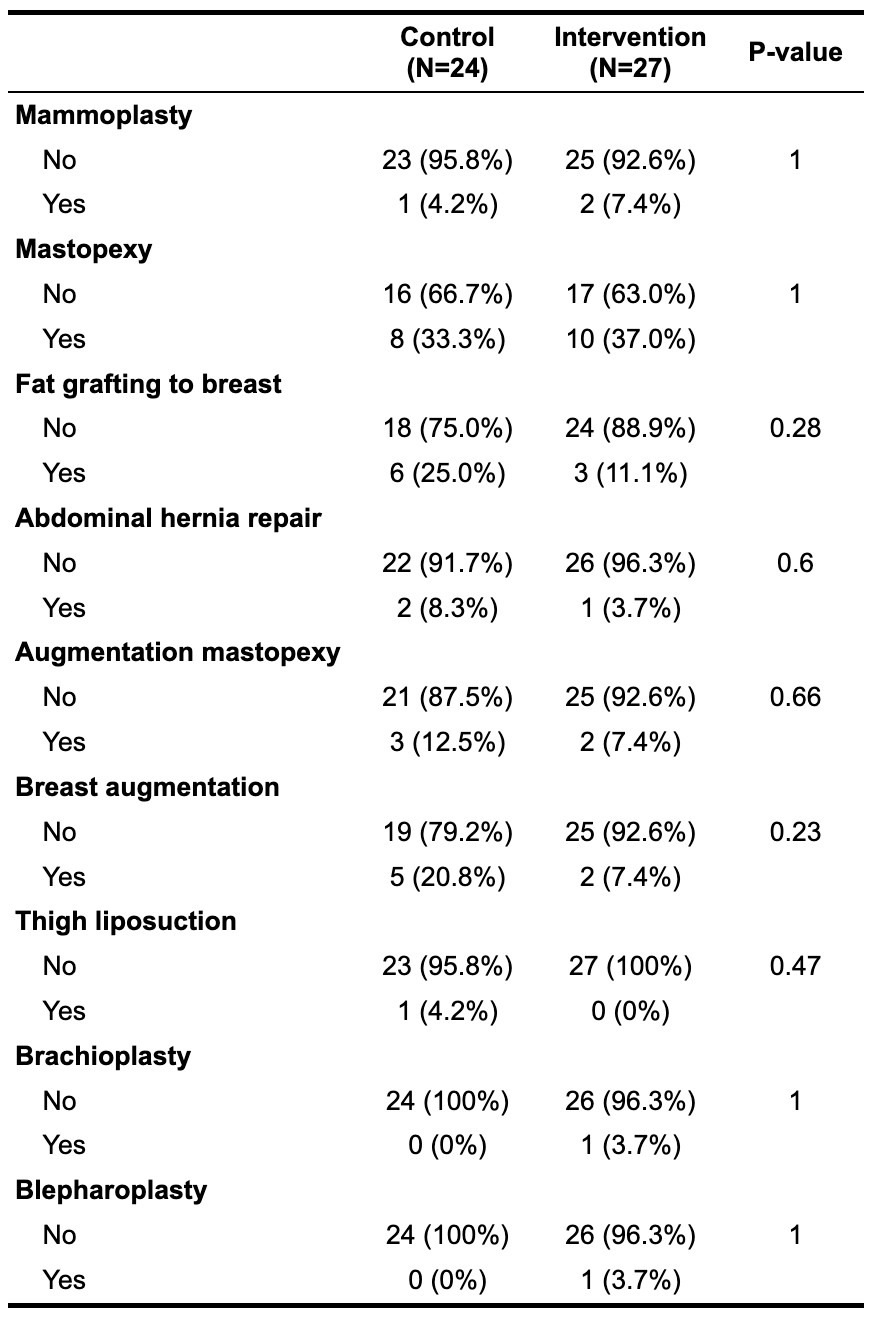


Supplementary Table 3. Concurrent procedures among patients who completed versus did not complete the study


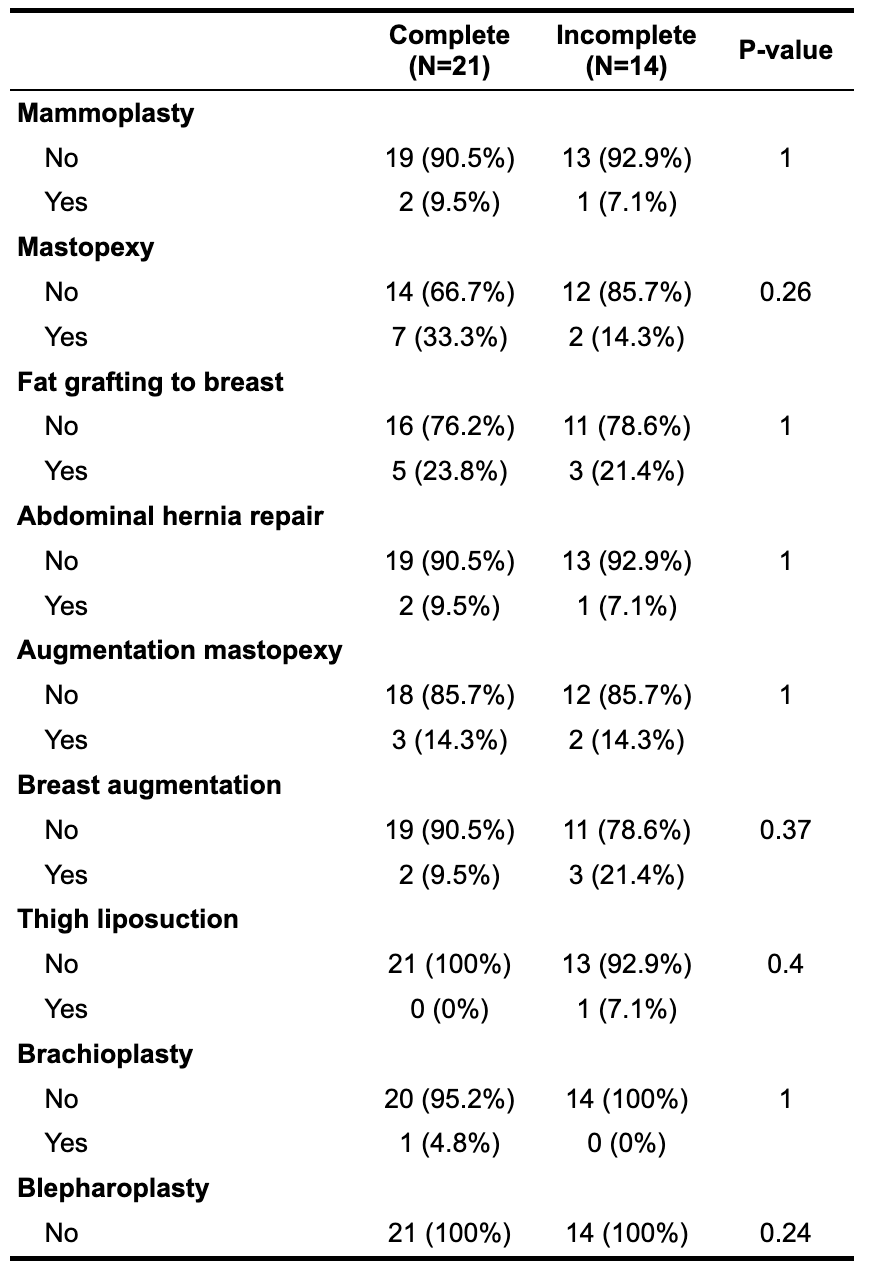

Supplement: ojaf135_Supplementary_Data [file ojaf135_supplementary_data.docx]
